# Estimating alcohol-related premature mortality in san francisco: use of population-attributable fractions from the global burden of disease study

**DOI:** 10.1186/1471-2458-10-682

**Published:** 2010-11-09

**Authors:** Brian S Katcher, Randy B Reiter, Tomás J Aragón

**Affiliations:** 1 Community Health Epidemiology, San Francisco Department of Public Health, San Francisco, California, USA; 2 Department of Clinical Pharmacy, School of Pharmacy, University of California, San Francisco, California, USA; 3 Division of Epidemiology, School of Public Health, University of California, Berkeley, California, USA; 4 Division of Preventive Medicine and Public Health, University of California, San Francisco, California, USA

## Abstract

**Background:**

In recent years, national and global mortality data have been characterized in terms of well-established risk factors. In this regard, alcohol consumption has been called the third leading "actual cause of death" (modifiable behavioral risk factor) in the United States, after tobacco use and the combination of poor diet and physical inactivity. Globally and in various regions of the world, alcohol use has been established as a leading contributor to the overall burden of disease and as a major determinant of health disparities, but, to our knowledge, no one has characterized alcohol-related harm in such broad terms at the local level. We asked how alcohol-related premature mortality in San Francisco, measured in years of life lost (YLLs), compares with other well-known causes of premature mortality, such as ischemic heart disease or HIV/AIDS.

**Methods:**

We applied sex- and cause-specific population-attributable fractions (PAFs) of years of life lost (YLLs) from the Global Burden of Disease Study to 17 comparable outcomes among San Francisco males and females during 2004-2007. We did this in three ways: Method 1 assumed that all San Franciscans drink like populations in developed economies. These estimates were limited to alcohol-related harm. Method 2 modified these estimates by including several beneficial effects. Method 3 assumed that Latino and Asian San Franciscans drink alcohol like populations in the global regions related to their ethnicity.

**Results:**

By any of these three methods, alcohol-related premature mortality accounts for roughly a tenth of all YLLs among males. Alcohol-related YLLs among males are comparable to YLLs for leading causes such as ischemic heart disease and HIV/AIDS, in some instances exceeding them. Latino and black males bear a disproportionate burden of harm. Among females, for whom estimates differed more by method and were smaller than those for males, alcohol-related YLLs are comparable to leading causes which rank somewhere between fifth and fourteenth.

**Conclusions:**

Alcohol consumption is a major contributor to premature mortality in San Francisco, especially among males. Interventions to avert alcohol-related harm in San Francisco should be taken at the population level and deserve the same attention that is given to other major risk factors, such as smoking or obesity.

## Background

Assessment is a core public health function [1]. At the local level, health assessments commonly include reportable infectious diseases, environmental health hazards, and local mortality data. In recent years, national and global mortality data have been characterized in terms of well-established risk factors [2-4]. In this regard, alcohol consumption has been called the third leading "actual cause of death" (modifiable behavioral risk factor) in the United States, after tobacco use and the combination of poor diet and physical inactivity [2,3]. Globally and in various regions of the world, alcohol use has been established as a major contributor to the overall burden of disease [5-9] and has been cited as a major determinant of health disparities in the United States [10-12]. However, to our knowledge, no one has characterized alcohol-related harm in such broad terms at the local level. Our study assessed the impact of alcohol consumption on premature mortality, measured in years of life lost (YLLs), among residents of the City and County of San Francisco, by sex and ethnicity during 2004-2007. We asked how alcohol-related premature mortality in San Francisco compares with other well-known causes of premature mortality, such as ischemic heart disease or HIV/AIDS.

Two-thirds of San Franciscans are current drinkers, compared with 55% of U.S. adults [13,14]. To minimize the risk of alcohol-related harm, U.S. Dietary Guidelines recommend either not drinking at all, or (unless prohibited by health status) limiting alcohol consumption to one drink per day for women and up to two drinks per day for men. One drink is defined as 5 fluid ounces of wine (148 ml), 12 ounces (355 ml) of beer, or 1.5 ounces (44 ml) of distilled spirits. These limits are not intended as an average over several days but rather as the amount consumed on any single day. Exceeding these limits increases the risk of health problems such as liver cirrhosis, hypertension, cancers of the upper gastrointestinal tract, injury, and violence. Among older adults, moderate alcohol consumption (defined by the above limits) is associated with the lowest all-cause and coronary heart disease mortality, though there are many other strategies for reducing cardiovascular risk; hence "it is not recommended that anyone begin drinking or drink more frequently on the basis of health considerations" [13].

The amount of alcohol consumed on any given day and the average volume of consumption are independent determinants of alcohol-related harm in populations [5,7]. Commonplace alcohol consumption patterns often fall outside of the limits set by U.S. Dietary Guidelines. Some drink more alcohol than this on most days. Some engage in fairly heavy alcohol consumption on at least an episodic basis. For example, according to the 2005 California Health Interview Survey, approximately one-fifth of San Franciscans engaged in binge drinking (5 or more drinks for males, 4 or more drinks for females) on at least one occasion during the past month [14].

Alcohol drinking patterns are culturally influenced (and therefore vary by ethnicity) [15], and San Francisco has a multi-ethnic population (approximately 48% white, 31% Asian, 10% Latino, and 8% black). Therefore, risky drinking patterns are more likely to occur in some segments of the population than others. For example, San Franciscans of Chinese descent drink alcohol infrequently or not at all [16,17]. On the other hand, most white San Franciscans do drink alcohol, and more than one-quarter of white San Franciscan drinkers engaged in binge drinking during the past month, according to the 2005 California Health Interview Survey--a higher proportion than among white drinkers in any other county in California [14].

Depending on how it is consumed, alcohol can contribute to many causes of premature mortality. For each cause of death to which alcohol use might contribute, various patterns of consumption are associated with various relative risks. If they are known (or reliably estimated), the relative risks and prevalence of drinking patterns associated with each of those risks can be used to calculate disease-specific population-attributable fractions (PAFs)--the fractions of cause-specific premature mortality that would have been avoided if exposure to the excess risk had been eliminated. Such an approach was recently used to estimate alcohol-related premature mortality in Ireland [8]. Unfortunately, most cities lack the resources to conduct this sort of an assessment, which requires detailed alcohol use survey data.

Therefore, we sought PAFs from other populations for application to our own mortality data, recognizing that PAFs are specific to the populations for which they were calculated [18-20]. Alcohol-related PAFs for the U.S. and individual states are available from the Centers for Disease Control and Prevention (CDC), and alcohol-related PAFs for various global regions are available from the Global Burden of Disease Study [6,7,21-27]. We used PAFs from the Global Burden of Disease Study, because they better suited our circumstances.

Although the CDC's Alcohol-Related Disease Impact (ARDI) software could be applied to a locality, this would require a mortality analysis using the groupings of ICD-10 codes that are particular to the ARDI software [21,22,27]. Such a project--an alcohol-burden-specific mortality analysis--would not place the estimated local alcohol-related burden within a larger context (e.g., relative to the leading causes of premature mortality) unless the results were compared with a separate, broader (all-cause) mortality analysis.

The purpose of this study was to estimate alcohol-related premature mortality in San Francisco, relative to the leading causes of premature mortality. We did this by applying methods from the Global Burden of Disease Study to our population. In 1996, the Global Burden of Disease Study established years of life lost (YLLs)--a population-based measure of premature mortality based on life expectancy at age of death--as a standard for health assessments [28], and the San Francisco Department of Public Health subsequently applied this metric to its own population, ranking the leading causes by sex and ethnicity [29-31]. The Global Burden of Disease Study's Comparative Risk Assessment Project systematically evaluated the changes in population health that would result from modifying the population distribution of exposure to major risk factors [6,32-35]. The Disease Control Priorities Project's most recent iteration of the Global Burden of Disease Study, published as the *Global Burden of Disease and Risk Factors *[4] builds upon the results from the Comparative Risk Assessment Project and includes PAFs for more than a dozen major risk factors in various regions of the world, including our own [6,35]. To assess alcohol-related burden, this most recent implementation of the Global Burden of Disease Study has established 17 sex- and cause-specific PAFs of YLLs for each global geographic and economic region. The use of externally-derived PAFs is problematic, because it assumes similar risks and exposures for populations that are potentially dissimilar. To minimize this problem, we used multiple approaches to estimate the impact of alcohol use on premature mortality in San Francisco as a kind of sensitivity analysis. Our goal was not a precise estimate but rather an assessment of order of magnitude of burden of disease from alcohol, relative to the magnitude of burden from Global Burden of Disease classifications of diseases.

## Methods

We calculated years of life lost (YLLs), stratified by sex, ethnicity, and underlying cause of death, using death registry data for San Francisco for four years, 2004 through 2007. Death data came from the annual death statistical master files prepared by the California Department of Public Center for Health Statistics, which include ICD-10 code for underlying cause of death. We included only deaths of San Francisco residents plus deaths occurring in San Francisco to persons with no other recorded place of residence. We used ICD-10 cause of death groupings from the Global Burden of Disease Study (GBD) [36]. Our listing of cause of death groupings dropped many used by GBD for tropical infectious diseases that do not occur in San Francisco.

YLLs were calculated using as life expectancy standards the Coale-Demeny Model Life Tables West for males (Level 25) and females (Level 26). Our method for interpolating years of life lost from the 21 age categories defined by these standard life tables has been described in detail elsewhere [29,31]. We did not apply discounting or age weighting to our YLL calculations. Total YLLs by cause were summed across age categories for each sex or sex and ethnicity stratum, and the leading causes contributing to burden of premature mortality (as measured by YLLs) were then ranked for males and females, and by ethnicity.

We based our study upon locally calculated YLLs rather than DALYs (disability adjusted life years), because DALYs are the sum of YLLs plus YLDs (years lived with disabilily), and YLDs cannot be measured directly for our population. Unfortunately, directly measuring YLDs is cost prohibitive and not practical for most local health jurisdictions.

We then multiplied these YLLs by population-attributable fractions (PAFs) for alcohol-related causes from Annex 4A of the *Global Burden of Disease and Risk Factors *[6] to estimate the burden of alcohol-related premature mortality, measured as years of life lost (YLLs). These PAFs are based upon estimates for six geographic regions of the developing world and two global economic regions (low- and middle-income countries, and high-income countries). Although alcohol use in San Francisco might be assumed to match that of high-income countries, the data on local alcohol use are too scant to support this assumption, and the City's ethnic variety (and, hence, drinking pattern variety) might call such an assumption into question. Therefore, we used multiple methods--each of which made its own set of assumptions about how alcohol is consumed in San Francisco--to produce a range of estimates of alcohol's impact on premature mortality in San Francisco. Each of these methods estimates alcohol-attributable YLLs by disease-specific cause and sums the results so that alcohol-attributable YLLs can be compared with YLLs for the various causes of premature mortality for male and female San Franciscans who died 2004-2007, overall and by ethnicity.

We applied the gender- and cause-specific alcohol-related population attributable fractions (PAFs) from the *Global Burden of Disease and Risk Factors *[6] as follows:

### Method 1 (harm only)

For Method 1 we assumed that alcohol use in San Francisco is the same as in high-income countries. These gender-specific alcohol-attributable fractions of alcohol's harmful effects and their application to cause-specific YLLs are shown in Tables 1 and 2. The calculated alcohol-attributable YLLs were then summed. This sum, alcohol-related harm, was then compared with YLLs for the leading causes of premature mortality by sex. This method assumes that alcohol use patterns are the same across all ethnicities. For this method we considered harm only, not benefits.

**Table 1:** Alcohol-related harm (method 1) among *males*, San Francisco 2004-2007

Rank	Cause	Deaths	YLL	PAF of YLL	YLL attributable to alcohol
1	Ischemic heart disease	2,023	25,604.3		
2	HIV/AIDS	519	17,570.9		
3	Violence	255	12,921.9	28%	3,618
4	Lung, bronchus, trachea cancers	813	12,760.4		
5	Drug overdose, unintentional	357	12,665.7	21%	2,660
6	Self-inflicted injuries	304	10,667.1	15%	1,600
7	Hypertensive heart disease	529	8,685.3	28%	2,432
8	Cerebrovascular disease	682	7,818.0	9%	704
9	Chronic obstructive pulm. dis.	541	6,492.4		
10	Alcohol use disorders	217	6,251.7	100%	6,252
11	Cirrhosis of the liver	205	5,448.8	60%	3,269
12	Lower respiratory infections	482	4,918.8		
13	Liver cancer	249	4,747.3	36%	1,709
14	Road traffic accidents	122	4,669.9	35%	1,634
15	Colon, rectum cancers	298	4,486.2		
				
*Other alcohol attributable causes:*					
	Falls		2,378.2	20%	476
	Low birthweight		1,760.0	2%	35
	Esophageal cancer		1,522.0	44%	670
	Mouth and oropharynx cancers		1,298.4	38%	493
	Drownings		1,128.2	24%	271
	Other neoplasms		870.6	10%	87
	Epilepsy		418.5	49%	205
	Unipolar depressive disorders		0	8%	0
				
All YLLs for SF males			225,369.9		
**Alcohol-attributable YLLs**					**26,115**
**% of YLLs attributable to alcohol**					**11.6%**

YLL (years of life lost) multiplied by PAF (population-attributable fraction) produces YLL attributable to alcohol. These results are for males (all ethnicities). See Additional file 1 for ethnic-specific rankings of causes and PAFs.

**Table 2:** Alcohol-related harm (method 1) among *females*, San Francisco 2004-2007

Rank	Cause	Deaths	YLL	PAF of YLL	YLL attributable to alcohol
1	Ischemic heart disease	1,938	17,365.7		
2	Cerebrovascular disease	1,007	9,866.2		
3	Lung, bronchus, trachea cancers	600	9,340.2		
4	Breast Cancer	383	7,653.7	9%	689
5	Hypertensive heart disease	518	5,603.2	21%	1,177
6	Alzheimer, other dementias	793	4,726.1		
7	Drug overdose, unintentional	112	4,482.0	17%	762
8	Lower respiratory infections	511	4,029.2		
9	Chronic obstructive pulmon. dis.	356	3,850.7		
10	Colon, rectum cancers	279	3,568.2		
11	Diabetes mellitus	244	3,088.7		
12	Self-inflicted injuries	84	3,088.7	10%	309
13	Pancreas cancer	199	3,019.9		
14	Ovarian cancer	139	2,639.1		
15	Road traffic accidents	68	2,541.5	16%	407
				
*Other alcohol-attributable causes:*					
	Cirrhosis of the liver		2,527.2	46%	1,163
	Liver cancer		2,031.5	27%	549
	Alcohol use disorders		1,393.3	100%	1,393
	Violence		1,350.6	27%	365
	Falls		1,165.4	8%	93
	Other neoplasms		1,099.3	7%	77
	Low birthweight		742.5	2%	15
	Mouth and oropharynx cancers		668.0	27%	180
	Esophageal cancer		422.6	36%	152
	Drownings		334.3	18%	60
	Epilepsy		226.8	35%	79
	Unipolar depressive disorders		38.9	2%	1
				
All YLLs for SF females			147,542.1		
**Alcohol-attributable YLLs**					**7,470**
**% of YLLs attributable to alcohol**					**5.1%**

YLL (years of life lost) multiplied by PAF (population-attributable fraction) produces YLL attributable to alcohol. These results are for females (all ethnicities). See Additional file 1 for ethnic-specific rankings of causes and PAFs.

### Method 2 (inclusion of beneficial effects)

Because alcohol use might have beneficial as well as harmful effects, we took the results from Method 1 and added the beneficial effects thought to be attributable to regular light to moderate alcohol consumption. These beneficial effects (a modest reduction in ischemic heart disease and diabetes mortality in both males and females, and a reduction in stroke mortality in females) have been demonstrated in some populations, and they are included in the alcohol-attributable fractions for high-income countries, but not for other Global Burden of Disease Study populations [6,7]. We applied them to all San Franciscans, regardless of ethnicity.

### Method 3 (ethnicity-adjusted drinking patterns)

Culture exerts a strong effect on drinking behavior. Drinking patterns in the United States vary widely by ethnicity, and San Francisco's population is ethnically diverse. Therefore, for Method 3 we linked ethnicity to global region, assuming that Asian San Franciscans drink alcohol like populations in East Asia and the Pacific, and that Latino San Franciscans drink like populations in Latin America and the Caribbean. Studies of drinking behaviors in the United States [15] and globally [7] lend support to this assumption. The gender-specific PAFs for these global regions are not the same as those shown in Tables 1 and 2 (see Additional file 1).

For whites, we used the alcohol-attributable fractions for high-income countries, including beneficial effects (Method 2 applied only to whites), because the epidemiological evidence for alcohol's beneficial effects comes from studies of mostly white volunteers, such as British Doctors [37], Nurses' Health Study participants [38,39], participants in the Health Professionals Follow-up Study [39-41], or American Cancer Study volunteers [42], who presumably drink small to moderate quantities on a regular basis. Non-white populations tend not to drink in this manner [15].

For African Americans, we used the alcohol-attributable fractions for high-income countries, but without beneficial effects (Method 1 applied only to African Americans), for two reasons: African American drinking patterns are more likely to be harmful than European American drinking patterns [15,43,44], and a beneficial effect does not appear to occur among African Americans [44,45], presumably because of drinking patterns.

## Results

### Method 1 (harm only)

As can be seen in Tables 1 and 2, the use of alcohol contributes to many causes of premature mortality, some of them among the leading causes. For males (Table 1), this list amounts to varying fractions of 17 different causes, the sum of which is 26,115 YLLs attributable to alcohol, or 11.6% of all the years of life lost by males in San Francisco during 2004-2007. This combined burden of alcohol-related YLLs exceeds the number of YLLs for ischemic heart disease, which is the leading cause. For females (Table 2), there are also 17 causes of alcohol-related harm, but--unlike males--cerebrovascular disease is not among them (because heavy drinking is less common among females), being replaced by breast cancer. There were 7,470 YLLs attributable to alcohol among San Francisco females, or 5.1% of all the years of life lost by females in San Francisco. This burden of alcohol-related YLLs exceeds the number of YLLs for hypertensive heart disease, which is the fifth leading cause. When this method is applied to each of four ethnic groups (Table 3), the harm falls disproportionately upon African-American and Latino males, for whom alcohol use accounts for an estimated 13.4% and 15.4% of premature mortality, respectively.

**Table 3:** Summary results, alcohol-related YLL as percentage of total YLL by demographic group, using three methods for estimating alcohol-related harm, San Francisco 2004-2007

Sex	Ethnicity	Method 1	Method 2	Method 3
***Male***	***Combined***	**11.6%**	**9.9%**	10.3%*
	Asian	8.5%	6.5%	7.3%
	African American	13.4%	12.1%	13.4%
	Latino	15.4%	14.3%	14.2%
	White	11.2%	9.4%	9.4%
***Female***	***Combined***	**5.1%**	**2.0%**	3.1%*
	Asian	3.6%	-0.6%	1.4%
	African American	5.3%	2.6%	5.3%
	Latino	5.6%	3.2%	5.5%
	White	5.4%	2.7%	2.7%

*The combined ethnicities for Method 3 are limited to the four that are shown, whereas the combined ethnicities for Methods 1 and 2 are based upon the entire population (e.g., Native Americans, mixed ethnicities, etc. are included).

See Additional file 1, which shows the data that were used to create this table.

### Method 2 (inclusion of beneficial effects)

Adding the beneficial effects of alcohol, and applying them to all San Franciscans, an estimated 3,746 YLLs from ischemic heart disease and diabetes would be avoided among males (Table 4), and an estimated 4,524 YLLs would be avoided among females (Table 5). When these avoided YLLs are subtracted from the tally of alcohol's harmful effects (Tables 1 and 2), the net alcohol impact on males is 22,369 YLLs (9.9% of all YLLs), and the net alcohol impact on females is 2,946 YLLs (2.0% of all YLLs). For males, this net impact of alcohol-related YLLs exceeds the number of YLLs for HIV/AIDS, which is the second leading cause of premature mortality. For females, this net impact of alcohol-related YLLs exceeds the number of YLLs for ovarian cancer, which is the fourteenth leading cause of premature mortality. When this method is applied to each of four ethnic groups (Table 3), the harm again falls disproportionately upon African-American and Latino males, for whom alcohol use accounts for an estimated 12.1% and 14.3% of premature mortality, respectively.

**Table 4:** Avoided and net harm from alcohol use (method 2) among *males*, San Francisco 2004-2007

		YLL	PAF	YLL attributableto alcohol
Alcohol-attributable harm, SF males (Table 1)				26,115
Benefits (based on all SF males):				
	Ischemic heart disease	25,604.3	-14%	-3,585
	Diabetes mellitus	4,038.3	-4%	-162
*total YLL avoided*				-3,746
**Net alcohol impact as YLL**				**22,369**
Alcohol YLL %*				**9.9%**

*Alcohol YLL % = YLL divided by YLL from all causes (225,369.9) for this demographic group

Method 2 modifies the harm shown in Table 1 by subtracting the beneficial effects that are presumed to be attributable to alcohol. This estimate is for all ethnicities combined.

**Table 5:** Avoided and net harm from alcohol use (method 2) among *females*, San Francisco 2004-2007

		YLL	PAF	YLL attributableto alcohol
Alcohol-attributable harm, SF females (Table 2)				7,470
Benefits (based on all SF females):				
	Ischemic heart disease	17,365.7	-10%	-1,737
	Cerebrovascular disease	9,866.2	-27%	-2,664
	Diabetes mellitus	3,088.7	-4%	-124
*total YLL avoided, females*				-4,524
**Net alcohol impact as YLL**				**2,946**
Alcohol YLL %**				**2.0%**

** Alcohol YLL % = YLL divided by YLL from all causes (147.592.1) for this demographic group

Method 2 modifies the harm shown in Table 2 by subtracting the beneficial effects that are presumed to be attributable to alcohol. This estimate is for all ethnicities combined.

### Method 3 (ethnicity-adjusted drinking patterns)

Using Method 3, which assumes a linkage between ethnicity in San Francisco and global region drinking patterns for Asians and Latinos and includes beneficial effects for whites but not blacks, alcohol use accounts for an estimated 10.3% of premature mortality among males, and 3.1% among females (Table 3). Unlike Methods 1 and 2, these estimates include only the four major ethnicities in San Francisco. The results for Latino males are consistent with those from other methods, despite the use of different PAFs (see Table 3 and Additional file 1).

## Discussion

The results from all three methods show that alcohol use, if considered as a separate cause of premature mortality, is comparable to the leading causes, particularly among males. Roughly one-tenth of YLLs can be attributed to alcohol use among males in San Francisco. Latino and African American males bear a disproportionate burden of harm. The fact that all three methods found similar results for males is striking.

Because YLLs are based on life expectancy, death at any age produces some premature mortality, but the effect is greater with some outcomes than with others (Tables 1 and 2). For example, the 255 deaths from violence among males produced 12,921.9 YLLs. On the other hand, the 529 deaths from hypertensive heart disease among males produced 8,685.3 YLLs. An important finding from this study is the wide distribution of alcohol-related harm. Alcohol is a contributor to premature mortality from injuries and from chronic diseases, as well as premature mortality directly attributable to alcoholism. Although these estimates are not precise, they are useful in broadly characterizing the impact of alcohol use on health in San Francisco.

The application of externally-derived PAFs creates some uncertainty in our results, but the ranges produced by the three methods lessen this problem in several ways: First, the assumption that beneficial cardiovascular effects might be applied to the entire population (Method 2) is weak, because some segments of the population are likely to drink in ways (e.g., regular heavy drinking, binge drinking) that contribute to cardiovascular disease mortality instead of preventing it [5,46-48]. Furthermore, large segments of the population (e.g., Asian females) drink alcohol too infrequently to gain any benefit. However, Method 2 provides a useful lower-boundary estimate of alcohol's net effect on San Francisco's population. It demonstrates that even the most optimistic estimate of alcohol's beneficial effects does little to offset the harmful effects of alcohol on the population as a whole, and on men in particular. Second, these estimates do not include still-to-be-established PAFs for HIV/AIDS (a leading cause of premature mortality among males in San Francisco) from alcohol-related unsafe sex, making them conservative. Finally, even if the lowest estimates (usually produced by Method 2) were further decreased by 50%, the alcohol-attributable YLLs would still rank among the top five causes for Latino and black males.

### Agreements/disagreements with prior studies

The disproportionate burden of alcohol-related premature mortality among males compared to females is well established [6,8,12,49]. As in previous mortality analyses of our population [29-31], the leading causes of premature mortality differed markedly among ethnic groups (see Additional file 1). The disparities we found in alcohol-related harm are a reflection of these rankings.

In studies of U.S. drinking, African Americans are more likely to be abstinent than whites, but those who do drink have higher average volumes of consumption and greater frequency of binge drinking. To a smaller extent, a similar trend exists among Latinos [15]. Heavy drinking has been declining among whites but not among African Americans or Latinos [50]. The disparities found in our study may reflect this trend.

### Strengths

This study has two major strengths:

First, it is based upon a comprehensive analysis of cause-specific premature mortality. This approach is useful for communicating the multiplicity of alcohol-related risks and for showing the burden of alcohol-related harm within the larger context of all-cause premature mortality. Moreover, it places the beneficial effects of alcohol within a larger population health perspective.

Second, there is a consistency of results with multiple methods, particularly among males.

### Limitations

This study also has a number of important limitations:

First, it makes assumptions about alcohol drinking patterns and average consumption among San Franciscans that may be erroneous. Only reliable local data can rectify this problem.

Second, some of the PAFs may not be appropriate to San Francisco's population, because the prevalence of competing risk factors (confounders) may not match those in the populations from which the PAFs were derived. Among these are infectious hepatitis (a risk for both liver cirrhosis and liver cancer) and illicit drugs (a risk for violence), both of which are highly prevalent in San Francisco. The net effects of these confounders are difficult to predict: Each of them might have led to an over-estimation of their respective alcohol-related outcomes (to the extent that these outcomes were the result of the competing risks alone and not due to alcohol), but alcohol potentiates the hepatotoxicity of infectious hepatitis and the likelihood of violence from cocaine or methamphetamine use, perhaps mitigating this over-estimation.

Third, the beneficial effects of alcohol are most prominent among older individuals, and alcohol-related injuries are more important among younger individuals. An age-segmented approach might have shown less net harm among older individuals.

Fourth, the ethnic groups used in this study were aggregates that may not have been appropriate. For example, Irish Americans and Italian Americans have similar average alcohol intakes, but the former are much more likely to binge drink. Another example of heterogeneity is Latinos of Mexican descent, who drink more heavily and are more prone to binge drink than those of Central American descent. Non-Japanese Asian Americans tend to be moderate drinkers [15]. In addition, ethnic drinking patterns may differ among first and subsequent generations of immigrants.

Fifth, while this estimation method is useful for characterizing the general local importance of alcohol to premature mortality, it would not be sensitive enough to monitor changes in these effects over time, particularly in response to interventions to reduce these effects.

And sixth, this study, which is limited to mortality outcomes, does not account for social harm, nor does it account for morbidity and attendant economic costs.

### Implications

The main implication of the study is that alcohol contributes to a wide array of causes of premature mortality, and by any of three methods, the sum of these various alcohol-related contributions to premature mortality in San Francisco is large. As such, this net effect should be considered as a major public health problem.

The World Health Organization's Expert Committee on Problems Related to Alcohol Consumption recently observed that

...implementing policy at the local level has a number of advantages. Local citizens are close to where the alcohol-related problems are experienced personally. The community must deal with injuries and deaths from road traffic accidents. Often it must provide hospital and emergency medical services, and provide interventions for people with alcohol use disorders. Alcohol problems are personal experiences for community members, and efforts to prevent or reduce future problems are also a personal matter. However, a common experience has been that local alcohol policy-making is hampered by restrictions on local action imposed by national or regional authorities [51].

Many of the most cost-effective mechanisms for averting alcohol-related problems [52] are policies that are set by the state. Among these are alcohol taxes [53,54], drinking-and-driving legislation, advertising bans, reduced hours of sale, and reduced density of retail outlets. The City and County of San Francisco has one of the highest concentrations of retail liquor outlets per 100,000 persons of any county in California [55]. Analyses such as this one are necessary for informing local advocacy to decrease the burden of alcohol-related disease.

## Conclusions

Alcohol use contributes to a wide array of causes of premature mortality. When alcohol's contribution to each of these causes is estimated in San Francisco and these various estimates are summed, alcohol use ranks among the leading causes of premature mortality. Interventions to avert alcohol-related harm in San Francisco should be made at the population level and deserve the same attention that is given to other major risk factors, such as smoking or obesity.

## Competing interests

The authors declare that they have no competing interests.

## Authors' contributions

RBR calculated the YLLs upon which the study is based. RBR and BSK reviewed the well-documented methods by which the Global Burden of Disease alcohol-related PAFs were created before applying them to local YLLs. TJA, who developed the methodology for calculating local YLLs, oversaw the study's design and implementation. BSK conceived and designed the study, conducted the analyses and literature reviews, and prepared the initial manuscript. All authors contributed substantially to the interpretation of findings and to the many manuscript revisions. All authors read and approved the final manuscript.

## Pre-publication history

The pre-publication history for this paper can be accessed here:

http://www.biomedcentral.com/1471-2458/10/682/prepub

## Supplementary Material

Additional file 1 **alcohol_yll.zip**. This is a mini-website, which provides supporting information. It is also posted at http://www.healthysf.org/alcohol_yll/. The website's pages were created from ten corresponding spreadsheets. Click here for file
