# Supplementary material for: Estimating alcohol-related premature mortality in san francisco: use of population-attributable fractions from the global burden of disease study
Source: BMC Public Health. 2010 Nov 9;10:682. doi: 10.1186/1471-2458-10-682 (PMC3091581; doi:10.1186/1471-2458-10-682)
Supplement: Additional file 1 — alcohol_yll.zip. This is a mini-website, which provides supporting information. It is also posted at http://www.healthysf.org/alcohol_yll/. The website's pages were created from ten corresponding spreadsheets. [file 1471-2458-10-682-S1.ZIP › alcohol_yll/asian_female_etoh.html]

Alcohol-Attributable YLLs


|  |  |  |  |  |  |  |  |  |  |  |
| --- | --- | --- | --- | --- | --- | --- | --- | --- | --- | --- |
| Asian female (San Francisco, 2004-07) alcohol-attributable YLLs by cause & method | | | | | | | | |  |  |
|  |  |  |  |  |  |  |  |  |  | **Other Depictions of Alcohol-related YLLs in San Francisco:**  SF females  SF males    **Asian females**  Asian males  Black females  Black Males  Latina females  Latino males  White females  White males    Home |
| *Sex/ethnic- specific rank* | *Specific cause of death* | *YLLs* | *PAF, Method 1: Harm only* | *PAF, Method 2: Includes an accounting of avoided harm* | *PAF, Method 3: Ethnicity as global region* | *YLL, Method 1: Harm only* | *YLL, Method 2: Includes an accounting of avoided harm* | *YLL, Method 3: Ethnicity as global region* |  |  |
| 1 | Ischemic heart disease | 4,561.8 |  | -10% |  |  | (456.2) |  |  |  |
| 2 | Cerebrovascular disease | 4,009.1 |  | -27% |  |  | (1,082.5) |  |  |  |
| 3 | Lung, bronchus, trachea cancers | 2,750.9 |  |  |  |  |  |  |  |  |
| 4 | Breast cancer | 1,977.2 | 9% | 9% | 3% | 177.9 | 177.9 | 59.3 |  |  |
| 5 | Hypertensive heart dis. | 1,393.9 | 21% | 21% | 6% | 292.7 | 292.7 | 83.6 |  |  |
| 6 | Colon, rectum cancers | 1,265.6 |  |  |  |  |  |  |  |  |
| 7 | Lower respiratory inf. | 1,239.0 |  |  |  |  |  |  |  |  |
| 8 | Alzheimer, other dementias | 1,110.1 |  |  |  |  |  |  |  |  |
| 9 | Diabetes mellitus | 1,074.8 |  | -4% | 0% |  | (43.0) |  |  |  |
| 10 | Road traffic accidents | 1,059.6 | 16% | 16% | 8% | 169.5 | 169.5 | 84.8 |  |  |
| 11 | Stomach cancer | 991.4 |  |  |  |  |  |  |  |  |
| 12 | Liver cancer | 975.8 | 27% | 27% | 9% | 263.5 | 263.5 | 87.8 |  |  |
| 13 | Pancreas cancer | 832.4 |  |  |  |  |  |  |  |  |
| 14 | Lymphomas, mult. myeloma | 717.9 |  |  |  |  |  |  |  |  |
| 15 | Ovary cancer | 602.3 |  |  |  |  |  |  |  |  |
|  |  |  |  |  |  |  |  |  |  |  |
| *Other alcohol-attributable causes:* | |  |  |  |  |  |  |  |  |  |
|  | Self-inflicted injuries | 559.8 | 10% | 10% | 5% | 56.0 | 56.0 | 28.0 |  |  |
|  | Falls | 422.7 | 8% | 8% | 5% | 33.8 | 33.8 | 21.1 |  |  |
|  | Other neoplasms | 305.9 | 7% | 7% | 2% | 21.4 | 21.4 | 6.1 |  |  |
|  | Mouth and oropharynx cancers | 303.6 | 27% | 27% | 9% | 82.0 | 82.0 | 27.3 |  |  |
|  | Violence | 236.4 | 27% | 27% | 15% | 63.8 | 63.8 | 35.5 |  |  |
|  | Low birthweight | 165.0 | 2% | 2% | 0% | 3.3 | 3.3 | - |  |  |
|  | Esophageal cancer | 134.1 | 36% | 36% | 13% | 48.3 | 48.3 | 17.4 |  |  |
|  | Cirrhosis of the liver | 133.7 | 46% | 46% | 7% | 61.5 | 61.5 | 9.4 |  |  |
|  | Drug overdose, unintentional | 100.8 | 17% | 17% | 7% | 17.1 | 17.1 | 7.1 |  |  |
|  | Drownings | 90.2 | 18% | 18% | 6% | 16.2 | 16.2 | 5.4 |  |  |
|  | Alcohol use disorders | 45.2 | 100% | 100% | 100% | 45.2 | 45.2 | 45.2 |  |  |
|  | Unipolar depressive disorders | 32.7 | 2% | 2% | 0% | 0.7 | 0.7 |  |  |  |
|  | Epilepsy | - | 35% | 35% | 6% |  |  |  |  |  |
|  |  |  |  |  |  |  |  |  |  |  |
| All YLLs for this demographic group | | 37,503.5 |  |  |  |  |  |  |  |  |
|  |  |  |  |  |  |  |  |  |  |  |
| Alcohol-attributable YLLs | |  |  |  |  | 1,353.0 | (228.6) | 518.0 |  |  |
|  |  |  |  |  |  |  |  |  |  |  |
| Percentage of YLLs attributable to alcohol | |  |  |  |  | 3.6% | -0.6% | 1.4% |  |  |
|  |  |  |  |  |  |  |  |  |  |  |
