# Supplementary material for: Estimating alcohol-related premature mortality in san francisco: use of population-attributable fractions from the global burden of disease study
Source: BMC Public Health. 2010 Nov 9;10:682. doi: 10.1186/1471-2458-10-682 (PMC3091581; doi:10.1186/1471-2458-10-682)
Supplement: Additional file 1 — alcohol_yll.zip. This is a mini-website, which provides supporting information. It is also posted at http://www.healthysf.org/alcohol_yll/. The website's pages were created from ten corresponding spreadsheets. [file 1471-2458-10-682-S1.ZIP › alcohol_yll/asian_male_etoh.html]

Alcohol-Attributable YLLs


|  |  |  |  |  |  |  |  |  |  |  |
| --- | --- | --- | --- | --- | --- | --- | --- | --- | --- | --- |
| Asian male (San Francisco, 2004-07) alcohol-attributable YLLs by cause & method | | | | | | |  |  |  |  |
|  |  |  |  |  |  |  |  |  |  | **Other Depictions of Alcohol-related YLLs in San Francisco:**  SF females  SF males    Asian females  **Asian males**  Black females  Black Males  Latina females  Latino males  White females  White males    Home |
| *Sex/ethnic- specific rank* | *Specific cause of death* | *YLLs* | *Method 1: Harm only* | *Method 2: Includes an accounting of avoided harm* | *Method 3: Ethnicity as global region* | *Method 1: Harm only* | *Method 2: Includes an accounting of avoided harm* | *Method 3: Ethnicity as global region* |  |
| 1 | Ischemic heart disease | 6,039.4 |  | -14% | 2% |  | (845.5) | 120.8 |  |
| 2 | Lung, bronchus, trachea cancers | 4,234.8 |  |  |  |  |  |  |  |
| 3 | Cerebrovascular disease | 3,024.0 | 9% | 9% | 13% | 272.2 | 272.2 | 393.1 |  |
| 4 | Liver cancer | 2,098.6 | 36% | 36% | 31% | 755.5 | 755.5 | 650.6 |  |
| 5 | Self-inflicted injuries, all mechanisms | 1,973.0 | 15% | 15% | 9% | 296.0 | 296.0 | 177.6 |  |
| 6 | Chronic obstructive pulmonary dis. | 1,700.4 |  |  |  |  |  |  |  |
| 7 | Hypertensive heart disease | 1,370.3 | 28% | 28% | 24% | 383.7 | 383.7 | 328.9 |  |
| 8 | Colon, rectum cancers | 1,306.8 |  |  |  |  |  |  |  |
| 9 | Lower respiratory infect. | 1,041.0 |  |  |  |  |  |  |  |
| 10 | Diabetes mellitus | 918.2 |  | -4% |  |  | (36.7) |  |  |
| 11 | Stomach cancer | 874.7 |  |  |  |  |  |  |  |
| 12 | Road traffic accidents | 860.5 | 35% | 35% | 23% | 301.2 | 301.2 | 197.9 |  |
| 13 | Lymphomas, mult. myeloma | 785.8 |  |  |  |  |  |  |  |
| 14 | Alzheimer, other dementias | 737.7 |  |  |  |  |  |  |  |
| 15 | Drug overdose, unintentional | 731.3 | 21% | 21% | 11% | 153.6 | 153.6 | 80.4 |  |
|  |  |  |  |  |  |  |  |  |  |
| *Other alcohol-attributable causes:* | |  |  |  |  |  |  |  |  |
|  | Violence/assault, all mechanisms | 624.4 | 28% | 28% | 18% | 174.8 | 174.8 | 112.4 |  |
|  | Mouth and oropharynx cancers | 590.4 | 38% | 38% | 31% | 224.4 | 224.4 | 183.0 |  |
|  | Falls | 549.2 | 20% | 20% | 12% | 109.8 | 109.8 | 65.9 |  |
|  | Cirrhosis of the liver | 506.7 | 60% | 60% | 45% | 304.0 | 304.0 | 228.0 |  |
|  | Low birthweight | 480.0 | 2% | 2% |  | 9.6 | 9.6 |  |  |
|  | Esophageal cancer | 446.0 | 44% | 44% | 39% | 196.2 | 196.2 | 173.9 |  |
|  | Alcohol use disorders | 377.7 | 100% | 100% | 100% | 377.7 | 377.7 | 377.7 |  |
|  | Other neoplasms | 233.2 | 10% | 10% | 7% | 23.3 | 23.3 | 16.3 |  |
|  | Epilepsy | 151.9 | 49% | 49% | 28% | 74.4 | 74.4 | 42.5 |  |
|  | Drownings | 110.6 | 24% | 24% | 8% | 26.5 | 26.5 | 8.8 |  |
|  | Unipolar depressive disorders | - | 8% | 8% | 2% |  |  |  |  |
|  |  |  |  |  |  |  |  |  |  |
| All YLLs for this demographic group | | 43,385.5 |  |  |  |  |  |  |  |
|  |  |  |  |  |  |  |  |  |  |
| Alcohol-attributable YLLs | |  |  |  |  | 3,682.9 | 2,800.7 | 3,158.0 |  |
|  |  |  |  |  |  |  |  |  |  |
| % of YLLs attributable to alcohol | |  |  |  |  | 8.5% | 6.5% | 7.3% |  |
