# Supplementary material for: Estimating alcohol-related premature mortality in san francisco: use of population-attributable fractions from the global burden of disease study
Source: BMC Public Health. 2010 Nov 9;10:682. doi: 10.1186/1471-2458-10-682 (PMC3091581; doi:10.1186/1471-2458-10-682)
Supplement: Additional file 1 — alcohol_yll.zip. This is a mini-website, which provides supporting information. It is also posted at http://www.healthysf.org/alcohol_yll/. The website's pages were created from ten corresponding spreadsheets. [file 1471-2458-10-682-S1.ZIP › alcohol_yll/black_females_etoh.html]

Alcohol-Attributable YLLs


|  |  |  |  |  |  |  |  |  |
| --- | --- | --- | --- | --- | --- | --- | --- | --- |
| Black female (San Francisco, 2004-07) alcohol-attributable YLLs by cause & method | | | | | | |  |  |
|  |  |  |  |  |  |  |  | **Other Depictions of Alcohol-related YLLs in San Francisco:**  SF females  SF males    Asian females  Asian males  **Black females**  Black Males  Latina females  Latino males  White females  White males    Home |
| *Sex/ethnic- specific rank* | *Specific cause of death* | *YLLs* | *Method 1: Harm only* | *Method 2: Includes an accounting of avoided harm* | *Method 1: Harm only* | *Method 2: Includes an accounting of avoided harm* |  |
| 1 | Ischemic heart disease | 2,753.2 |  | -10% |  | (275.3) |  |
| 2 | Lung, bronchus, trachea cancers | 1,577.1 |  |  |  |  |  |
| 3 | Drug overdose, unintentional | 1,576.3 | 17% | 17% | 268.0 | 268.0 |  |
| 4 | Cerebrovascular disease | 1,512.7 |  | -27% |  | (408.4) |  |
| 5 | Hypertensive heart disease | 1,498.6 | 21% | 21% | 314.7 | 314.7 |  |
| 6 | HIV/AIDS | 1,163.8 |  |  |  |  |  |
| 7 | Breast cancer | 956.2 | 9% | 9% | 86.1 | 86.1 |  |
| 8 | Diabetes mellitus | 821.5 |  | -4% |  | -32.9 |  |
| 9 | Drug use disorders | 704.1 |  |  |  |  |  |
| 10 | Colon, rectum cancers | 663.3 |  |  |  |  |  |
| 11 | Chronic obstructive pulmonary dis. | 613.2 |  |  |  |  |  |
| 12 | Nephritis and nephrosis | 612.1 |  |  |  |  |  |
| 13 | Violence | 528.4 | 27% | 27% | 142.7 | 142.7 |  |
| 14 | Birth asphyxia, trauma | 495.0 |  |  |  |  |  |
| 15 | Alzheimer, other dementias | 493.2 |  |  |  |  |  |
|  |  |  |  |  |  |  |  |
| *Other alcohol-related causes:* | | |  |  |  |  |  |
|  | Cirrhosis of the liver | 408.9 | 46% | 46% | 188.1 | 188.1 |  |
|  | Liver cancer | 331.2 | 27% | 27% | 89.4 | 89.4 |  |
|  | Low birthweight | 247.5 | 2% | 2% | 5.0 | 5.0 |  |
|  | Road traffic accidents | 198.2 | 16% | 16% | 31.7 | 31.7 |  |
|  | Alcohol use disorders | 180.2 | 100% | 100% | 180.2 | 180.2 |  |
|  | Other neoplasms | 142.9 | 7% | 7% | 10.0 | 10.0 |  |
|  | Liver cancer | 136.1 | 35% | 35% | 47.6 | 47.6 |  |
|  | Falls | 85.6 | 8% | 8% | 6.8 | 6.8 |  |
|  | Esophageal cancer | 61.5 | 36% | 36% | 22.1 | 22.1 |  |
|  | Self-inflicted injuries | 53.3 | 10% | 10% | 5.3 | 5.3 |  |
|  | Drownings | 34.0 | 18% | 18% | 6.1 | 6.1 |  |
|  | Mouth and oropharynx cancers | 6.2 | 27% | 27% | 1.7 | 1.7 |  |
|  | Unipolar depressive disorders | - | 2% | 2% |  |  |  |
|  |  |  |  |  |  |  |  |
| All YLLs for this demographic group | | 26,342.3 |  |  |  |  |  |
|  |  |  |  |  |  |  |  |
| Alcohol-attributable YLLs | |  |  |  | 1,405.5 | 688.9 |  |
|  |  |  |  |  |  |  |  |
| % of YLLs attributable to alcohol | |  |  |  | 5.3% | 2.6% |  |
