# Supplementary material for: Estimating alcohol-related premature mortality in san francisco: use of population-attributable fractions from the global burden of disease study
Source: BMC Public Health. 2010 Nov 9;10:682. doi: 10.1186/1471-2458-10-682 (PMC3091581; doi:10.1186/1471-2458-10-682)
Supplement: Additional file 1 — alcohol_yll.zip. This is a mini-website, which provides supporting information. It is also posted at http://www.healthysf.org/alcohol_yll/. The website's pages were created from ten corresponding spreadsheets. [file 1471-2458-10-682-S1.ZIP › alcohol_yll/black_male_etoh.html]

Alcohol-Attributable YLLs


|  |  |  |  |  |  |  |  |  |
| --- | --- | --- | --- | --- | --- | --- | --- | --- |
| Black male (San Francisco, 2004-07) alcohol-attributable YLLs by cause & method | | | | | | |  |  |
|  |  |  |  |  |  |  |  | **Other Depictions of Alcohol-related YLLs in San Francisco:**  SF females  SF males    Asian females  Asian males  Black females  **Black Males**  Latina females  Latino males  White females  White males    Home |
| *Sex/ethnic- specific rank* | *Specific cause of death* | *YLLs* | *Method 1: Harm only* | *Method 2: Includes an accounting of avoided harm* | *Method 1: Harm only* | *Method 2: Includes an accounting of avoided harm* |  |
| 1 | Violence/assault, all mechanisms | 7,313.6 | 28% | 28% | 2,047.8 | 2,047.8 |  |
| 2 | Ischemic heart disease | 3,840.8 |  | -14% |  | (537.7) |  |
| 3 | HIV/AIDS | 3,342.0 |  |  |  |  |  |
| 4 | Drug overdose, unintentional | 3,186.1 | 21% | 21% | 669.1 | 669.1 |  |
| 5 | Hypertensive heart disease | 2,016.9 | 28% | 28% | 564.7 | 564.7 |  |
| 6 | Lung, bronchus, trachea cancers | 1,912.9 |  |  |  |  |  |
| 7 | Drug use disorders | 1,363.0 |  |  |  |  |  |
| 8 | Cerebrovascular disease | 1,302.5 | 9% | 9% | 117.2 | 117.2 |  |
| 9 | Chronic obstructive pulmonary dis. | 1,234.0 |  |  |  |  |  |
| 10 | Alcohol use disorders | 1,116.6 | 100% | 100% | 1,116.6 | 1,116.6 |  |
| 11 | Diabetes mellitus | 973.3 |  | -4% |  | (38.9) |  |
| 12 | Inflammatory heart disease | 892.1 |  |  |  |  |  |
| 13 | Self-inflicted injuries, all mechanisms | 838.3 | 15% | 15% | 125.7 | 125.7 |  |
| 14 | Road traffic accidents | 801.9 | 35% | 35% | 280.7 | 280.7 |  |
| 15 | Cirrhosis of the liver | 706.0 | 60% | 60% | 423.6 | 423.6 |  |
|  |  |  |  |  |  |  |  |
| *Other alcohol-attributable causes:* | |  |  |  |  |  |  |
|  | Liver cancer | 526.6 | 36% | 36% | 189.6 | 189.6 |  |
|  | Falls | 331.4 | 20% | 20% | 66.3 | 66.3 |  |
|  | Mouth and oropharynx cancers | 180.5 | 38% | 38% | 68.6 | 68.6 |  |
|  | Drownings | 177.8 | 24% | 24% | 42.7 | 42.7 |  |
|  | Other neoplasms | 136.5 | 10% | 10% | 13.7 | 13.7 |  |
|  | Esophageal cancer | 123.5 | 44% | 44% | 54.3 | 54.3 |  |
|  | Low birthweight | 80.0 | 2% | 2% | 1.6 | 1.6 |  |
|  | Epilepsy | 62.9 | 49% | 49% | 30.8 | 30.8 |  |
|  | Unipolar depressive disorders | - | 8% | 8% | - | - |  |
|  |  |  |  |  |  |  |  |
| All YLLs for this demographic group | | 43,335.6 |  |  |  |  |  |
|  |  |  |  |  |  |  |  |
| Alcohol-attributable YLLs | |  |  |  | 5,813.0 | 5,236.3 |  |
|  |  |  |  |  |  |  |  |
| % of YLLs attributable to alcohol | |  |  |  | 13.4% | 12.1% |  |
