# Supplementary material for: Estimating alcohol-related premature mortality in san francisco: use of population-attributable fractions from the global burden of disease study
Source: BMC Public Health. 2010 Nov 9;10:682. doi: 10.1186/1471-2458-10-682 (PMC3091581; doi:10.1186/1471-2458-10-682)
Supplement: Additional file 1 — alcohol_yll.zip. This is a mini-website, which provides supporting information. It is also posted at http://www.healthysf.org/alcohol_yll/. The website's pages were created from ten corresponding spreadsheets. [file 1471-2458-10-682-S1.ZIP › alcohol_yll/index.html]

alcohol-related premaure mortality in SF


**Alcohol-attributable YLLs
by cause & method, San Francisco, 2004-2007**

SF
females

SF males

\_\_\_\_\_\_\_\_\_

Asian
females

Asian
males

Black
females

Black
Males

Latina
females

Latino
males

White
females

White
males

This mini-website provides supporting
information for "Estimating alcohol-related premature mortality in San
Francisco: use of population-attributable fractions from the Global Burden of
Disease Study."

For more information contact brian
[at] healthysf.org

This page was updated on July 5,
2010
