# Supplementary material for: Estimating alcohol-related premature mortality in san francisco: use of population-attributable fractions from the global burden of disease study
Source: BMC Public Health. 2010 Nov 9;10:682. doi: 10.1186/1471-2458-10-682 (PMC3091581; doi:10.1186/1471-2458-10-682)
Supplement: Additional file 1 — alcohol_yll.zip. This is a mini-website, which provides supporting information. It is also posted at http://www.healthysf.org/alcohol_yll/. The website's pages were created from ten corresponding spreadsheets. [file 1471-2458-10-682-S1.ZIP › alcohol_yll/latina_female_etoh.html]

Alcohol-Attributable YLLs


|  |  |  |  |  |  |  |  |  |  |  |
| --- | --- | --- | --- | --- | --- | --- | --- | --- | --- | --- |
| Latina female (San Francisco, 2004-07) alcohol-attributable YLLs by cause & method | | | | | | |  |  |  |  |
|  |  |  |  |  |  |  |  |  |  | **Other Depictions of Alcohol-related YLLs in San Francisco:**  SF females  SF males    Asian females  Asian males  Black females  Black Males  **Latina females**  Latino males  White females  White males    Home |
| *Sex/ethnic- specific rank* | *Specific cause of death* | *YLLs* | *Method 1: Harm only* | *Method 2: Includes an accounting of avoided harm* | *Method 3: Ethnicity as global region* | *Method 1: Harm only* | *Method 2: Includes an accounting of avoided harm* | *Method 3: Ethnicity as global region* |  |
| 1 | Ischemic heart disease | 1,340.7 |  | -10% | 2% |  | (134.1) | 26.8 |  |
| 2 | Cerebrovascular disease | 751.0 |  | -27% | 7% |  | (202.8) | 52.6 |  |
| 3 | Cirrhosis of the liver | 633.1 | 46% | 46% | 42% | 291.2 | 291.2 | 265.9 |  |
| 4 | Breast cancer | 554.2 | 9% | 9% | 7% | 49.9 | 49.9 | 38.8 |  |
| 5 | Road traffic accidents | 553.9 | 16% | 16% | 14% | 88.6 | 88.6 | 77.5 |  |
| 6 | Lung, bronchus, trachea cancers | 491.0 |  |  |  |  |  |  |  |
| 7 | Pancreas cancer | 487.4 |  |  |  |  |  |  |  |
| 8 | Leukemia | 444.2 |  |  |  |  |  |  |  |
| 9 | Hypertensive heart disease | 399.7 | 21% | 21% | 19% | 83.9 | 83.9 | 75.9 |  |
| 10 | Alzheimer, other dementias | 358.2 |  |  |  |  |  |  |  |
| 11 | Lower respiratory inf. | 348.0 |  |  |  |  |  |  |  |
| 12 | Congenital anomalies | 331.5 |  |  |  |  |  |  |  |
| 13 | Self-inflicted injuries | 325.4 | 10% | 10% | 9% | 32.5 | 32.5 | 29.3 |  |
| 14 | Drug overdose, unintentional | 325.1 | 17% | 17% | 11% | 55.3 | 55.3 | 35.8 |  |
| 15 | Diabetes mellitus | 308.1 |  | -4% | 0% |  | (12.3) |  |  |
|  |  |  |  |  |  |  |  |  |  |
| *Other alcohol-attributable causes:* | |  |  |  |  |  |  |  |  |
|  | Liver cancer | 289.3 | 27% | 27% | 24% | 78.1 | 78.1 | 69.4 |  |
|  | Violence | 102.1 | 27% | 27% | 27% | 27.6 | 27.6 | 27.6 |  |
|  | Other neoplasms | 83.7 | 7% | 7% | 6% | 5.9 | 5.9 | 5.0 |  |
|  | Low birthweight | 82.5 | 2% | 2% | 3% | 1.7 | 1.7 | 2.5 |  |
|  | Epilepsy | 81.8 | 35% | 35% | 28% | 28.6 | 28.6 | 22.9 |  |
|  | Falls | 77.8 | 8% | 8% | 7% | 6.2 | 6.2 | 5.4 |  |
|  | Esophageal cancer | 47.6 | 36% | 36% | 33% | 17.1 | 17.1 | 15.7 |  |
|  | Mouth and oropharynx cancers | 34.0 | 27% | 27% | 26% | 9.2 | 9.2 | 8.8 |  |
|  | Alcohol use disorders | 24.8 | 100% | 100% | 100% | 24.8 | 24.8 | 24.8 |  |
|  | Unipolar depressive disorders | 6.2 | 2% | 2% | 1% | 0.1 | 0.1 | 0.1 |  |
|  | Drownings | - | 18% | 18% | 10% |  |  |  |  |
|  |  |  |  |  |  |  |  |  |  |
| All YLLs for this demographic group | | 14,217.8 |  |  |  |  |  |  |  |
|  |  |  |  |  |  |  |  |  |  |
| Alcohol-attributable YLLs | |  |  |  |  | 800.8 | 451.6 | 784.9 |  |
|  |  |  |  |  |  |  |  |  |  |
| Percentage of YLLs attributable to alcohol | |  |  |  |  | 5.6% | 3.2% | 5.5% |  |
