# Supplementary material for: Estimating alcohol-related premature mortality in san francisco: use of population-attributable fractions from the global burden of disease study
Source: BMC Public Health. 2010 Nov 9;10:682. doi: 10.1186/1471-2458-10-682 (PMC3091581; doi:10.1186/1471-2458-10-682)
Supplement: Additional file 1 — alcohol_yll.zip. This is a mini-website, which provides supporting information. It is also posted at http://www.healthysf.org/alcohol_yll/. The website's pages were created from ten corresponding spreadsheets. [file 1471-2458-10-682-S1.ZIP › alcohol_yll/latino_male_etoh.html]

Alcohol-Attributable YLLs


|  |  |  |  |  |  |  |  |  |  |  |
| --- | --- | --- | --- | --- | --- | --- | --- | --- | --- | --- |
| **Latino male (San Francisco, 2004-07) alcohol-attributable YLLs by cause & method** | | | | | | |  |  |  |  |
|  |  |  |  |  |  |  |  |  |  | **Other Depictions of Alcohol-related YLLs in San Francisco:**  SF females  SF males    Asian females  Asian males  Black females  Black Males  Latina females  **Latino males**  White females  White males    Home |
| *Sex/ethnic- specific rank* | *Specific cause of death* | *YLLs* | *Method 1: Harm only* | *Method 2: Includes an accounting of avoided harm* | *Method 3: Ethnicity as global region* | *Method 1: Harm only* | *Method 2: Includes an accounting of avoided harm* | *Method 3: Ethnicity as global region* |  |
| 1 | Violence/assault, all mech. | 3,043.7 | 28% | 28% | 43% | 852.2 | 852.2 | 1,308.8 |  |
| 2 | HIV/AIDS | 2,605.8 |  |  |  | - | - | - |  |
| 3 | Ischemic heart disease | 1,978.8 |  | -14% | 14% |  | (277.0) | 277.0 |  |
| 4 | Cirrhosis of the liver | 1,247.8 | 60% | 60% | 54% | 748.7 | 748.7 | 673.8 |  |
| 5 | Drug overdose, unintentional | 1,208.1 | 21% | 21% | 27% | 253.7 | 253.7 | 326.2 |  |
| 6 | Road traffic accidents | 1,167.8 | 35% | 35% | 47% | 408.7 | 408.7 | 548.9 |  |
| 7 | Self-inflicted injuries, all mech. | 991.4 | 15% | 15% | 25% | 148.7 | 148.7 | 247.9 |  |
| 8 | Alcohol use disorders | 953.8 | 100% | 100% | 100% | 953.8 | 953.8 | 953.8 |  |
| 9 | Cerebrovascular disease | 900.0 | 9% | 9% | 12% | 81.0 | 81.0 | 108.0 |  |
| 10 | Low birthweight | 640.0 | 2% | 2% | 3% | 12.8 | 12.8 | 19.2 |  |
| 11 | Hypertensive heart disease | 597.4 | 28% | 28% | 26% | 167.3 | 167.3 | 155.3 |  |
| 12 | Diabetes mellitus | 518.2 |  | -4% |  |  | (20.7) | - |  |
| 13 | Lung, bronchus, trachea cancers | 459.6 |  |  |  |  |  | - |  |
| 14 | Drug use disorders | 399.2 |  |  |  | - | - | - |  |
| 15 | Lymphomas, mult. myeloma | 375.7 |  |  |  | - | - | - |  |
|  |  |  |  |  |  |  |  |  |  |
| *Other alcohol-attributable causes:* | |  |  |  |  | - |  |  |  |
|  | Liver cancer | 299.4 | 36% | 36% | 32% | 107.8 | 107.8 | 95.8 |  |
|  | Esophageal cancer | 182.7 | 44% | 44% | 43% | 80.4 | 80.4 | 78.6 |  |
|  | Falls | 122.5 | 20% | 20% | 30% | 24.5 | 24.5 | 36.8 |  |
|  | Drownings | 121.5 | 24% | 24% | 32% | 29.2 | 29.2 | 38.9 |  |
|  | Epilepsy | 86.2 | 49% | 49% | 37% | 42.2 | 42.2 | 31.9 |  |
|  | Mouth, oropharynx cancers | 46.6 | 38% | 38% | 36% | 17.7 | 17.7 | 16.8 |  |
|  | Other neoplasms | 25.5 | 10% | 10% | 8% | 2.6 | 2.6 | 2.0 |  |
|  | Unipolar depressive disorders | - | 8% | 8% | 7% | - | - | - |  |
|  |  |  |  |  |  |  |  |  |  |
| All YLLs for this demographic group |  | 25,490.9 |  |  |  |  |  |  |  |
|  |  |  |  |  |  |  |  |  |  |
| Alcohol-attributable YLLs | |  |  |  |  | 3,931.3 | 3,633.5 | 3,610.8 |  |
|  |  |  |  |  |  |  |  |  |  |
| % of YLLs attributable to alcohol |  |  |  |  |  | 15.4% | 14.3% | 14.2% |  |
