# Supplementary material for: Estimating alcohol-related premature mortality in san francisco: use of population-attributable fractions from the global burden of disease study
Source: BMC Public Health. 2010 Nov 9;10:682. doi: 10.1186/1471-2458-10-682 (PMC3091581; doi:10.1186/1471-2458-10-682)
Supplement: Additional file 1 — alcohol_yll.zip. This is a mini-website, which provides supporting information. It is also posted at http://www.healthysf.org/alcohol_yll/. The website's pages were created from ten corresponding spreadsheets. [file 1471-2458-10-682-S1.ZIP › alcohol_yll/sf_female_etoh.html]

Alcohol-Attributable YLLs


|  |  |  |  |  |  |  |  |  |
| --- | --- | --- | --- | --- | --- | --- | --- | --- |
| San Francisco female (all ethnicities, 2004-07) alcohol-attributable YLLs by cause & method | | | | | | |  |  |
|  |  |  |  |  |  |  |  | **Other Depictions of Alcohol-related YLLs in San Francisco:**  **SF females**  SF males    Asian females  Asian males  Black females  Black Males  Latina females  Latino males  White females  White males    Home |
| *Sex/ethnic- specific rank* | *Specific cause of death* | *YLLs* | *Method 1: Harm only* | *Method 2: Includes an accounting of avoided harm* | *Method 1: Harm only* | *Method 2: Includes an accounting of avoided harm* |  |
| 1 | Ischemic heart disease | 17,365.7 |  | -10% |  | (1736.6) |  |
| 2 | Cerebrovascular disease | 9,866.2 |  | -27% |  | (2663.9) |  |
| 3 | Lung, bronchus, trachea cancers | 9,340.2 |  |  |  |  |  |
| 4 | Breast Cancer | 7,653.7 | 9% | 9% | 688.8 | 688.8 |  |
| 5 | Hypertensive heart disease | 5,603.2 | 21% | 21% | 1,176.7 | 1176.7 |  |
| 6 | Alzheimer, other dementias | 4,726.1 |  |  |  |  |  |
| 7 | Drug overdose, unintentional | 4,482.0 | 17% | 17% | 761.9 | 761.9 |  |
| 8 | Lower respiratory infections | 4,029.2 |  |  |  |  |  |
| 9 | Chronic obstructive pulmonary dis. | 3,850.7 |  |  |  |  |  |
| 10 | Colon, rectum cancers | 3,568.2 |  |  |  |  |  |
| 11 | Diabetes mellitus | 3,088.7 |  | -4% |  | (123.5) |  |
| 12 | Self-inflicted injuries | 3,088.7 | 10% | 10% | 308.9 | 308.9 |  |
| 13 | Pancreas cancer | 3,019.9 |  |  |  |  |  |
| 14 | Ovary cancer | 2,639.1 |  |  |  |  |  |
| 15 | Road traffic accidents | 2,541.5 | 16% | 16% | 406.6 | 406.6 |  |
|  |  |  |  |  |  |  |  |
| *Other alcohol-attributable causes:* | |  |  |  |  |  |  |
|  | Cirrhosis of the liver | 2,527.2 | 46% | 46% | 1,162.5 | 1162.5 |  |
|  | Liver cancer | 2,031.5 | 27% | 27% | 548.5 | 548.5 |  |
|  | Alcohol use disorders | 1,393.3 | 100% | 100% | 1,393.3 | 1393.3 |  |
|  | Violence | 1,350.6 | 27% | 27% | 364.7 | 364.7 |  |
|  | Falls | 1,165.4 | 8% | 8% | 93.2 | 93.2 |  |
|  | Other neoplasms | 1,099.3 | 7% | 7% | 77.0 | 77.0 |  |
|  | Low birthweight | 742.5 | 2% | 2% | 14.9 | 14.9 |  |
|  | Mouth and oropharynx cancers | 668.0 | 27% | 27% | 180.4 | 180.4 |  |
|  | Esophageal cancer | 422.6 | 36% | 36% | 152.1 | 152.1 |  |
|  | Drownings | 334.3 | 18% | 18% | 60.2 | 60.2 |  |
|  | Epilepsy | 226.8 | 35% | 35% | 79.4 | 79.4 |  |
|  | Unipolar depressive disorders | 38.9 | 2% | 2% | 0.8 | 0.8 |  |
|  |  |  |  |  |  |  |  |
| All YLLs for this demographic group | | 147,542.1 |  |  |  |  |  |
|  |  |  |  |  |  |  |  |
| Alcohol-attributable YLLs | |  |  |  | 7,469.8 | 2,945.8 |  |
|  |  |  |  |  |  |  |  |
| % of YLLs attributable to alcohol | |  |  |  | 5.1% | 2.0% |  |
