# Supplementary material for: Estimating alcohol-related premature mortality in san francisco: use of population-attributable fractions from the global burden of disease study
Source: BMC Public Health. 2010 Nov 9;10:682. doi: 10.1186/1471-2458-10-682 (PMC3091581; doi:10.1186/1471-2458-10-682)
Supplement: Additional file 1 — alcohol_yll.zip. This is a mini-website, which provides supporting information. It is also posted at http://www.healthysf.org/alcohol_yll/. The website's pages were created from ten corresponding spreadsheets. [file 1471-2458-10-682-S1.ZIP › alcohol_yll/sf_male_etoh.html]

Alcohol-Attributable YLLs


|  |  |  |  |  |  |  |  |  |
| --- | --- | --- | --- | --- | --- | --- | --- | --- |
| San Francisco male (all ethnicities, 2004-07) alcohol-attributable YLLs by cause & method | | | | | | |  |  |
|  |  |  |  |  |  |  |  | **Other Depictions of Alcohol-related YLLs in San Francisco:**  SF females  **SF males**    Asian females  Asian males  Black females  Black Males  Latina females  Latino males  White females  White males    Home |
| *Sex/ethnic- specific rank* | *Specific cause of death* | *YLLs* | *Method 1: Harm only* | *Method 2: Includes an accounting of avoided harm* | *Method 1: Harm only* | *Method 2: Includes an accounting of avoided harm* |  |
| 1 | Ischemic heart disease | 25,604.3 |  | -14% |  | (3,584.6) |  |
| 2 | HIV/AIDS | 17,570.9 |  |  |  |  |  |
| 3 | Violence | 12,921.9 | 28% | 28% | 3,618.1 | 3,618.1 |  |
| 4 | Lung, bronchus, trachea cancers | 12,760.4 |  |  |  |  |  |
| 5 | Drug overdose, unintentional | 12,665.7 | 21% | 21% | 2,659.8 | 2,659.8 |  |
| 6 | Self-inflicted injuries | 10,667.1 | 15% | 15% | 1,600.1 | 1,600.1 |  |
| 7 | Hypertensive heart disease | 8,685.3 | 28% | 28% | 2,431.9 | 2,431.9 |  |
| 8 | Cerebrovascular disease | 7,818.0 | 9% | 9% | 703.6 | 703.6 |  |
| 9 | Chronic obstructive pulmonary dis. | 6,492.4 |  |  |  |  |  |
| 10 | Alcohol use disorders | 6,251.7 | 100% | 100% | 6,251.7 | 6,251.7 |  |
| 11 | Cirrhosis of the liver | 5,448.8 | 60% | 60% | 3,269.3 | 3,269.3 |  |
| 12 | Lower respiratory inf. | 4,918.8 |  |  |  |  |  |
| 13 | Liver cancer | 4,747.3 | 36% | 36% | 1,709.0 | 1,709.0 |  |
| 14 | Road traffic accidents | 4,669.9 | 35% | 35% | 1,634.5 | 1,634.5 |  |
| 15 | Colon, rectum cancers | 4,486.2 |  |  |  |  |  |
|  |  |  |  |  |  |  |  |
| *Other alcohol attributable causes:* | |  |  |  |  |  |  |
|  | Diabetes mellitus | 4,038.3 |  | -4% |  | (161.5) |  |
|  | Falls | 2,378.2 | 20% | 20% | 475.6 | 475.6 |  |
|  | Low birthweight | 1,760.0 | 2% | 2% | 35.2 | 35.2 |  |
|  | Esophageal cancer | 1,522.0 | 44% | 44% | 669.7 | 669.7 |  |
|  | Mouth, oropharynx cancers | 1,298.4 | 38% | 38% | 493.4 | 493.4 |  |
|  | Drownings | 1,128.2 | 24% | 24% | 270.8 | 270.8 |  |
|  | Other neoplasms | 870.6 | 10% | 10% | 87.1 | 87.1 |  |
|  | Epilepsy | 418.5 | 49% | 49% | 205.1 | 205.1 |  |
|  | Unipolar depressive disorders | - | 8% | 8% |  |  |  |
|  |  |  |  |  |  |  |  |
| All YLLs for this demographic group | | 225,370 |  |  |  |  |  |
|  |  |  |  |  |  |  |  |
| Alcohol-attributable YLLs | |  |  |  | 26,114.8 | 22,368.6 |  |
|  |  |  |  |  |  |  |  |
| % of YLLs attributable to alcohol | |  |  |  | 11.6% | 9.9% |  |
