# Supplementary material for: Estimating alcohol-related premature mortality in san francisco: use of population-attributable fractions from the global burden of disease study
Source: BMC Public Health. 2010 Nov 9;10:682. doi: 10.1186/1471-2458-10-682 (PMC3091581; doi:10.1186/1471-2458-10-682)
Supplement: Additional file 1 — alcohol_yll.zip. This is a mini-website, which provides supporting information. It is also posted at http://www.healthysf.org/alcohol_yll/. The website's pages were created from ten corresponding spreadsheets. [file 1471-2458-10-682-S1.ZIP › alcohol_yll/white_female_etoh.html]

Alcohol-Attributable YLLs


| White female (San Francisco, 2004-07) alcohol-attributable YLLs by cause & method | | | | | | |  |  |
|  |  |  |  |  |  |  |  | **Other Depictions of Alcohol-related YLLs in San Francisco:**  SF females  SF males    Asian females  Asian males  Black females  Black Males  Latina females  Latino males  **White females**  White males    Home |
| *Sex/ethnic- specific rank* | *Specific cause of death* | *YLLs* | *Method 1: Harm only* | *Method 2: Includes an accounting of avoided harm* | *Method 1: Harm only* | *Method 2: Includes an accounting of avoided harm* |  |
| 1 | Ischemic heart disease | 8,307.4 |  | -10.0% |  | (830.7) |  |
| 2 | Lung, bronchus, trachea cancers | 4,399.3 |  |  |  |  |  |
| 3 | Breast cancer | 4,050.5 | 9% | 9% | 364.5 | 364.5 |  |
| 4 | Cerebrovascular disease | 3,419.2 |  | -27% |  | (923.2) |  |
| 5 | Alzheimer, other dementias | 2,760.6 |  |  |  |  |  |
| 6 | Drug overdose, unintentional | 2,446.0 | 17% | 17% | 415.8 | 415.8 |  |
| 7 | Chronic obstructive pulmonary disease | 2,443.8 |  |  |  |  |  |
| 8 | Hypertensive heart disease | 2,227.0 | 21% | 21% | 467.7 | 467.7 |  |
| 9 | Self-inflicted injuries | 2,048.7 | 10% | 10% | 204.9 | 204.9 |  |
| 10 | Lower respiratory inf. | 2,014.3 |  |  |  |  |  |
| 11 | Ovary cancer | 1,438.1 |  |  |  |  |  |
| 12 | Colon, rectum cancers | 1,367.8 |  |  |  |  |  |
| 13 | Cirrhosis of the liver | 1,317.7 | 46% | 46% | 606.1 | 606.1 |  |
| 14 | Pancreas cancer | 1,178.5 |  |  |  |  |  |
| 15 | Lymphomas, mult. myeloma | 1,030.7 |  |  |  |  |  |
|  |  |  |  |  |  |  |  |
| *Other alcohol-attributable causes:* | |  |  |  |  |  |  |
|  | Alcohol use disorders | 885.8 | 100% | 100% | 885.8 | 885.8 |  |
|  | Diabetes mellitus | 859.3 |  | -4% |  | (34.4) |  |
|  | Road traffic accidents | 725.1 | 16% | 16% | 116.0 | 116.0 |  |
|  | Falls | 579.3 | 8% | 8% | 46.3 | 46.3 |  |
|  | Other neoplasms | 566.7 | 7% | 7% | 39.7 | 39.7 |  |
|  | Liver cancer | 414.8 | 27% | 27% | 112.0 | 112.0 |  |
|  | Violence | 401.8 | 27% | 27% | 108.5 | 108.5 |  |
|  | Mouth and oropharynx cancers | 315.2 | 27% | 27% | 85.1 | 85.1 |  |
|  | Low birthweight | 247.5 | 2% | 2% | 5.0 | 5.0 |  |
|  | Drownings | 210.2 | 18% | 18% | 37.8 | 37.8 |  |
|  | Esophageal cancer | 179.3 | 36% | 36% | 64.5 | 64.5 |  |
|  | Epilepsy | 8.9 | 35% | 35% | 3.1 | 3.1 |  |
|  | Unipolar depressive disorders | - | 2% | 2% |  |  |  |
|  |  |  |  |  |  |  |  |
| All YLLs for this demographic group | | 66,479.1 |  |  |  |  |  |
|  |  |  |  |  |  |  |  |
| Alcohol-attributable YLLs | |  |  |  | 3,562.9 | 1,774.6 |  |
|  |  |  |  |  |  |  |  |
| % of YLLs attributable to alcohol | |  |  |  | 5.4% | 2.7% |  |
