# Supplementary material for: Estimating alcohol-related premature mortality in san francisco: use of population-attributable fractions from the global burden of disease study
Source: BMC Public Health. 2010 Nov 9;10:682. doi: 10.1186/1471-2458-10-682 (PMC3091581; doi:10.1186/1471-2458-10-682)
Supplement: Additional file 1 — alcohol_yll.zip. This is a mini-website, which provides supporting information. It is also posted at http://www.healthysf.org/alcohol_yll/. The website's pages were created from ten corresponding spreadsheets. [file 1471-2458-10-682-S1.ZIP › alcohol_yll/white_male_etoh.html]

Alcohol-Attributable YLLs


|  |  |  |  |  |  |  |  |  |
| --- | --- | --- | --- | --- | --- | --- | --- | --- |
| White male (San Francisco, 2004-07) alcohol-attributable YLLs by cause & method | | | | | | |  |  |
|  |  |  |  |  |  |  |  | **Other Depictions of Alcohol-related YLLs in San Francisco:**  SF females  SF males    Asian females  Asian males  Black females  Black Males  Latina females  Latino males  White females  **White males**    Home |
| *Sex/ethnic- specific rank* | *Specific cause of death* | *YLLs* | *Method 1: Harm only* | *Method 2: Includes an accounting of avoided harm* | *Method 1: Harm only* | *Method 2: Includes an accounting of avoided harm* |  |
| 1 | Ischemic heart disease | 13,379.3 |  | -14% |  | (1,873.1) |  |
| 2 | HIV/AIDS | 10,396.7 |  |  |  |  |  |
| 3 | Drug overdose, unintentional | 7,108.9 | 21% | 21% | 1,492.9 | 1492.9 |  |
| 4 | Self-inflicted injuries, all mech. | 6,368.7 | 15% | 15% | 955.3 | 955.3 |  |
| 5 | Lung, bronchus, trachea cancers | 5,898.5 |  |  |  |  |  |
| 6 | Hypertensive heart disease | 4,532.7 | 28% | 28% | 1,269.2 | 1269.2 |  |
| 7 | Alcohol use disorders | 3,700.2 | 100% | 100% | 3,700.2 | 3700.2 |  |
| 8 | Chronic obstructive pulmonary dis. | 3,241.0 |  |  |  |  |  |
| 9 | Lower respiratory infections | 2,934.6 |  |  |  |  |  |
| 10 | Cirrhosis of the liver | 2,863.5 | 60% | 60% | 1,718.1 | 1718.1 |  |
| 11 | Cerebrovascular dis. | 2,427.8 | 9% | 9% | 218.5 | 218.5 |  |
| 12 | Colon, rectum cancers | 2,251.7 |  |  |  |  |  |
| 13 | Drug use disorders | 2,061.1 |  |  |  |  |  |
| 14 | Liver cancer | 1,791.3 | 36% | 36% | 644.9 | 644.9 |  |
| 15 | Road traffic accidents | 1,702.9 | 35% | 35% | 596.0 | 596.0 |  |
|  |  |  |  |  |  |  |  |
| *Other alcohol attributable causes:* |  |  |  |  |  |  |  |
|  | Diabetes mellitus | 1,514.4 |  | -4% |  | (60.6) |  |
|  | Violence/assault, all mechanisms | 1,322.0 | 28% | 28% | 370.2 | 370.2 |  |
|  | Falls, unintentional | 1,318.9 | 20% | 20% | 263.8 | 263.8 |  |
|  | Esophagus cancer | 752.3 | 44% | 44% | 331.0 | 331.0 |  |
|  | Drownings, unintentional/unknown | 718.3 | 24% | 24% | 172.4 | 172.4 |  |
|  | Low birthweight | 480.0 | 2% | 2% | 9.6 | 9.6 |  |
|  | Mouth, oropharynx cancers | 423.3 | 38% | 38% | 160.9 | 160.9 |  |
|  | Other neoplasms | 417.8 | 10% | 10% | 41.8 | 41.8 |  |
|  | Epilepsy | 117.5 | 49% | 49% | 57.6 | 57.6 |  |
|  | Unipolar depressive disorders | - | 8% | 8% |  |  |  |
|  |  |  |  |  |  |  |  |
| All YLLs for this demographic group | | 107,262.6 |  |  |  |  |  |
|  |  |  |  |  |  |  |  |
| Alcohol-attributable YLLs | |  |  |  | 12,002.2 | 10,068.5 |  |
|  |  |  |  |  |  |  |  |
| % of YLLs attributable to alcohol | |  |  |  | 11.2% | 9.4% |  |
